# Use of mHealth in promoting maternal and child health in “BIMARU” states of India “A health system strengthening strategy”: Systematic literature review

**DOI:** 10.1371/journal.pdig.0000403

**Published:** 2024-02-02

**Authors:** Khushbu Singh, Matthew R. Walters

**Affiliations:** 1 College of Medical, Veterinary & Life Sciences, University of Glasgow, Glasgow, Scotland, United Kingdom; 2 School of Medicine, Dentistry & Nursing, University of Glasgow, Glasgow, Scotland, United Kingdom; Iran University of Medical Sciences, IRAN (ISLAMIC REPUBLIC OF)

## Abstract

Low-middle income countries like India bear a heavier burden of maternal, childcare, and child mortality rates when compared with high-income countries, which highlights the disparity in global health. Numerous societal, geopolitical, economic, and institutional issues have been linked to this inequality. mHealth has the potential to ameliorate these challenges by providing health services and health-related information with the assistance of frontline workers in the provision of prepartum, delivery, and postnatal care to improve maternal and child health outcomes in hard-to-reach areas in low- and middle-income countries (LMICs). However, there is limited evidence to support how mHealth can strengthen maternal and child health in India. The scoping review guideline in the Cochrane Handbook was used to retrieve studies from 4 international databases: CINAHL, Embase, Medline Ovid, and PubMed. This search strategy used combined keywords (MeSH terms) related to maternal and child healthcare, mHealth, and BIMARU in conjunction with database-controlled vocabulary. Out of 278 records, 8 publications were included in the review. The included articles used mHealth for data collection, eLearning, communication, patient monitoring, or tracking to deliver maternal and neonatal care. The results of these papers reflected a favourable effect of mHealth on the target population and found that it altered their attitudes and behaviours about healthcare. Higher job satisfaction and self-efficiency were reported by mHealth user care providers. Multiple barriers to the acceptance of mHealth exist, but the majority of the evidence points towards the feasibility of the intervention in a clinical setting. The mHealth has positive potential for improving maternal and child health outcomes in low-resource settings in India’s BIMARU states by strengthening the healthcare system. The results of the study could be used in the tailoring of an effective mHealth intervention and implementation strategy in a similar context. However, there is a need for economic evaluation in the future to bridge the knowledge gap regarding the cost-effectiveness of mHealth interventions.

## Introduction

Maternal mortality is considered one of the most challenging healthcare situations globally. In 2017, approximately 810 women died every day from a preventable cause associated with pregnancy and childbirth, which amounts to 295,000 total deaths that year [1]. The World Health Organization (WHO) estimates that the global maternal mortality rate (MMR) decreased by 35% from the year 2000 through 2017 from an estimated 451,000 total deaths in 2000, which corresponds to an annual reduction in the MMR of 2.9%. [1].

Despite a positive global decline in the MMR, a large number of low- and-middle-income countries (LMICs) continue to report a higher incidence of maternal fatalities [2], among which Nigeria and India are top on the list and accounted for approximately one-third of estimated global maternal deaths (67,000 and 35,000, respectively) in 2017 [3]. According to Amoakoh-Coleman and colleagues and Tunçalp and colleagues, use of innovative technologies in delivering maternal healthcare services, particularly during intrapartum period, showed potential in reducing the MMR [3,4].

While the existing literature shows promising results in the realm of maternal and child health outcomes with mHealth intervention. The fundamental marker of an effective mHealth technology is to enhance healthcare delivery in a way that is both scalable and sustainable from a payer perspective, as well as acceptable and cost-effective to the stakeholders.

The globalisation of mobile phone technology, with almost 100% of the world’s population covered by mobile signals, made it a potential intervention for addressing reproductive, maternal, new-born care, and child health (RMNCH) outcomes in LMICs [4]. The mobile devices used for improving health outcomes, health-related research, and healthcare services are often called “mHealth,” which is a proliferation of mobile innovation [5].

According to Blaya, Fraser, and Holt, mHealth improves the efficiency of frontline health workers (FLWs), particularly those who provide primary care in resource-limited settings [6]. mHealth offers various potential benefits over traditional paper-based methods with positive outcomes in the investigated parameters, like facilitating medical data collection, eLearning, disease prevention, and patient self-management [6–8]. It facilitates medical professionals’ performance and decision-making capabilities in the context of digitalized data and increases access to cost-effective, modern healthcare in rural areas of LMIC [6,7,9]. Furthermore, mHealth also supports and sustains beneficiaries’ adherence to medical recommendations that enhance the effectiveness of care providers and strengthen intrapartum and postpartum care [7,10].

### mHealth in India

India is the second-most populated country in the world, and approximately 80% of the Indian population lives in rural areas with access to 25% of healthcare infrastructure [11]. Providing healthcare to such a large population with unequal health-resource distribution is an immense challenge, so the question is whether mHealth could help in overcome these challenges and accelerate progress towards health equity [9].

**Fig 1:**
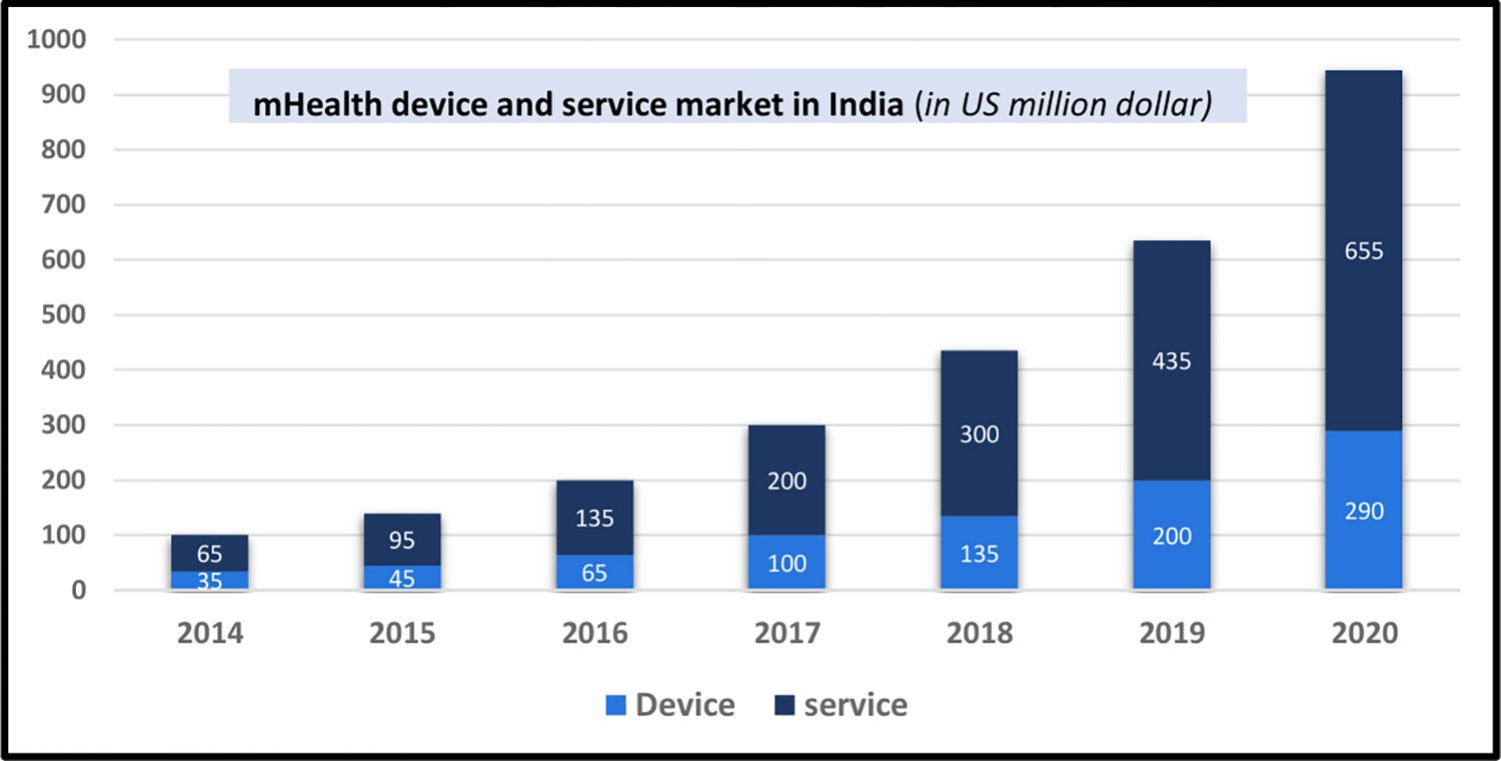
Size of mHealth device and services in India’s market (in US million dollar). **(A)** The bar graph represents the market size of the mHealth (mobile health) device and service market in India from the year 2014 to the year 2020 (these values are round figures have been rounded. **(B)** The y-axis represents the market size in US million dollars, while the x-axis represents the years. **(C)** The light blue bars represent the market size of mHealth devices, while the dark blue bars represent the market size of mHealth services.

India is experiencing unpredicted growth in the field of information and communication technology, which has resulted in increased tele-density (Fig 1) and improved mobile phone usage even in rural areas [9,12]. Hence, mHealth could be an appropriate need-based and cost-effective technology to strengthen this weakened health systems by improving health-related outcomes through enhancing health services accessibility, quality, knowledge, behaviour, and experience of beneficiaries and care providers [13,14].

### What are BIMARU states?

**Fig 2:**
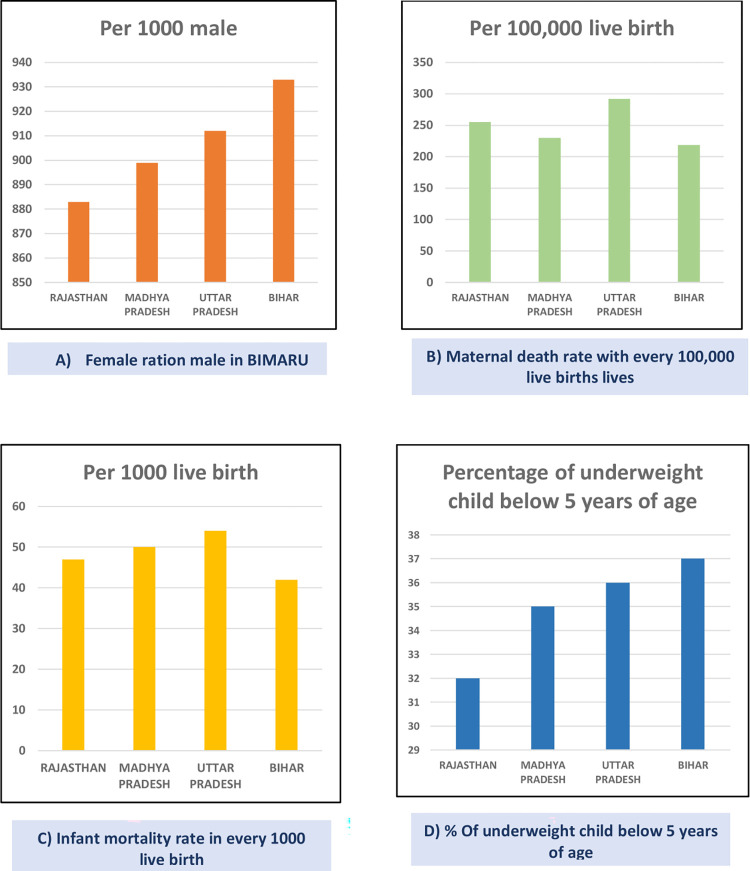
Analytical data from BIMARU states of India. **(A)** The graphs represent various health indicators in BIMARU states of India based on the Indian Government’s annual health surveys conducted between 2011 and 2013. Each graph focuses on a specific health indicator and displays the data for BIMARU states. The y-axis represents the respective indicator, while the x-axis represents the BIMARU states. Each bar in the legend represents the data related to the specific indicator for the BIMARU states. **(B)** Graph A: The graph represents the Female-to-Male Ratio in BIMARU states. The x-axis represents the BIMARU states, and the y-axis represents the Female-to-Male Ratio. The data highlights the Female-to-Male Ratio for each state, with Rajasthan having the lowest ratio of 883 females per 1,000 males, followed by Madhya Pradesh (899:1,000), Uttar Pradesh (912:1,000), and Bihar (933:1,000). **(C)** Graph B: The graph represents the Maternal Death Rate due to Complications in BIMARU states. The x-axis represents the BIMARU states, and the y-axis represents the Maternal Death Rate per 100,000 births. Each bar in the legend represents the Maternal Death Rate due to Complications for the respective state, with Rajasthan with 255, Madhya Pradesh with 230, Uttar Pradesh with 292, and Bihar with 219 deaths per 100,000 births. **(D)** Graph C: The graph represents the Newborn/Infant Mortality Rate in BIMARU states. The x-axis represents the BIMARU states, and the y-axis represents the Mortality Rate per 1,000 births. Each bar in the legend represents the Newborn/Infant Mortality Rate for the respective state, with Rajasthan having 47 deaths, Madhya Pradesh 50 deaths, Uttar Pradesh with 54 deaths, and Bihar with 42 deaths per 1,000 births. **(E)** Graph D: The graph represents the percentage of undernourished/underweight children below 5 years in BIMARU states (Rajasthan, Madhya Pradesh, Uttar Pradesh, and Bihar). The x-axis represents the BIMARU states, and the y-axis represents the percentage of undernourished/underweight children. Each bar in the legend represents the percentage for the respective state, with Rajasthan having 32% undernourished or underweight children, Madhya Pradesh with 35%, Uttar Pradesh with 36%, and Bihar with 37%.

“BIMARU” is an acronym coined by Ashish Bose in the 1980s by combining the initial letters of the Indian states of Bihar, Madhya Pradesh, Rajasthan, and Uttar Pradesh [15]. The word “BIMARU” is similar to the Hindi word “Bimar,” which means “sick.” These states are classified as the most backward in terms of mortality and health infrastructure, consistently display poor performance in a number of socioeconomic aspects [15]. Furthermore, in the Indian Government’s annual health surveys between 2011 and 2013, BIMARU states were ranked among the worst child health states and were later named one of the High Priority States to Promote RMNCH (other contributing factors for this low health indicator achievement are shown in Fig 2) [15–17].

### Gap in knowledge

The existing literature shows promising results of mHealth interventions in the realm of maternal and child health outcomes, but there are 2 major gaps in this nascent area. First, limited evidence of mHealth impact in large-scale and complex healthcare settings like India points towards a knowledge gap regarding health outcomes and the variables that influence them, such as access, quality, and experience [13,18]. Second, to date, no systematic literature review has been carried out to assess how mHealth has strengthened the maternal and childcare systems in India’s BIMARU states. Hence, the rationale of this systematic literature review is to evaluate the best available evidence regarding the effectiveness of mHealth interventions to improve maternal and child health outcomes in the BIMARU states of India in order to address the knowledge gap.

## Methodology

This systematic review of the literature was conducted in accordance with the requirements of the Preferred Reporting Items for Systematic Reviews and Meta-Analyses (PRISMA) [19] and the Cochrane Handbook [20], both of which offer direction toward a thorough and accurate literature review methodology.

### Eligibility criteria

Eligibility criteria were developed, and terms for the exclusion and inclusion of studies are listed in Table 1.

**Table 1:** Eligibility criteria.

Inclusion criteria	Exclusion criteria
• Place: BIMARU states of India (Bi: Bihar, MA: Madhya Pradesh, R: Rajasthan and U: Uttar Pradesh) • Study focused on pregnancy, maternity care, infant, and/or childcare • Implementation of any mHealth or mobile application in the study • Used primary or secondary data with a method of selection	• Studies conducted before 2012 and after 2022 • Articles not published in English • Only technological or economically focused study • Published as editorial, a book, a letter, unpublished research, and abstracts suggesting future studies

### Search strategy and information source

Four international electronic databases—CINAHL, Embase, MEDLINE, and PubMed were searched systemically using keywords and MeSH terms and search string of each database is demonstrated in S1 Appendix [21]. The reference list was evaluated by 2 reviewers apart from the authors to assess the papers’ eligibility for inclusion.

Manually searched studies from the reference lists were not included, as stated in the Cochrane Handbook: “positive studies are more likely to be cited” and “retrieving literature by scanning reference lists may thus produce a biased sample of studies” [20]. There was no limitation placed on the circumstances that qualified for inclusion, and both qualitative and quantitative studies were taken into consideration.

### PICOT formation of research question

“How do the effectiveness and feasibility of mHealth interventions, and the challenges encountered in their acceptability among all stakeholders, compare to traditional paper-based or non-technological methods to improve healthcare in BIMARU states of India from 2012 to 2022?”

### Study selection and data extraction

Using the reference management programme Endnote, search results from all databases were imported, producing 278 articles. One group was created for all articles, and separate groups were created for each database search result. Articles, on the other hand, were not eliminated by collecting both theoretical and empirical data from published studies using a critical appraisal tool or a technical quality basis.

### Quality assessment

The quality of each included study was assessed by using a suitable critical appraisal checklist in accordance with the research study design (S1 Appendix), Critical Appraisal Skills Programme (CASP) [22] for randomised control trials and qualitative studies; Joanna Briggs Institute (JBI) checklist for quasi-experiments and non-randomised study designs [23]; and Critical Appraisal Framework for Original Research (CAFFOR) for mixed-method studies [24]. Despite the fact that the results varied depending on the research design, the following characteristics were the focus of all these checklist tools: clarity in aim and objective, appropriateness of methodology, appropriateness of study design in addressing objectives of research (internal validity), recruitment and data collection and analysis strategies (external validity), consideration of ethical issues, reporting quality, clarity in the finding statement, and value of the research (transferability and generalisability) [22–24].

### Data collection and synthesis

The included studies’ results provided heterogenous data, which constrained the qualitative synthesis of the data. Therefore, a descriptive data synthesis was employed to better understand the research question related to mHealth in the target group. First, initial data from the included papers was extracted and coded in accordance with the research question. In the following stage, these codes were categorised into related subject areas and combined to form descriptive themes [25]. The names of the authors, the publication year, the state, the mHealth intervention, the intended audience, and the results were extracted. The source of the evidence and an assessment of the evidence for the included research were described. The last phase was a critical analysis of the outcomes extracted from the themes by interacting, comparing, and contrasting the findings to draw conclusions. Afterward, a recommendation was made in light of this analysis [26].

## Results

### Study selected

**Fig 3:**
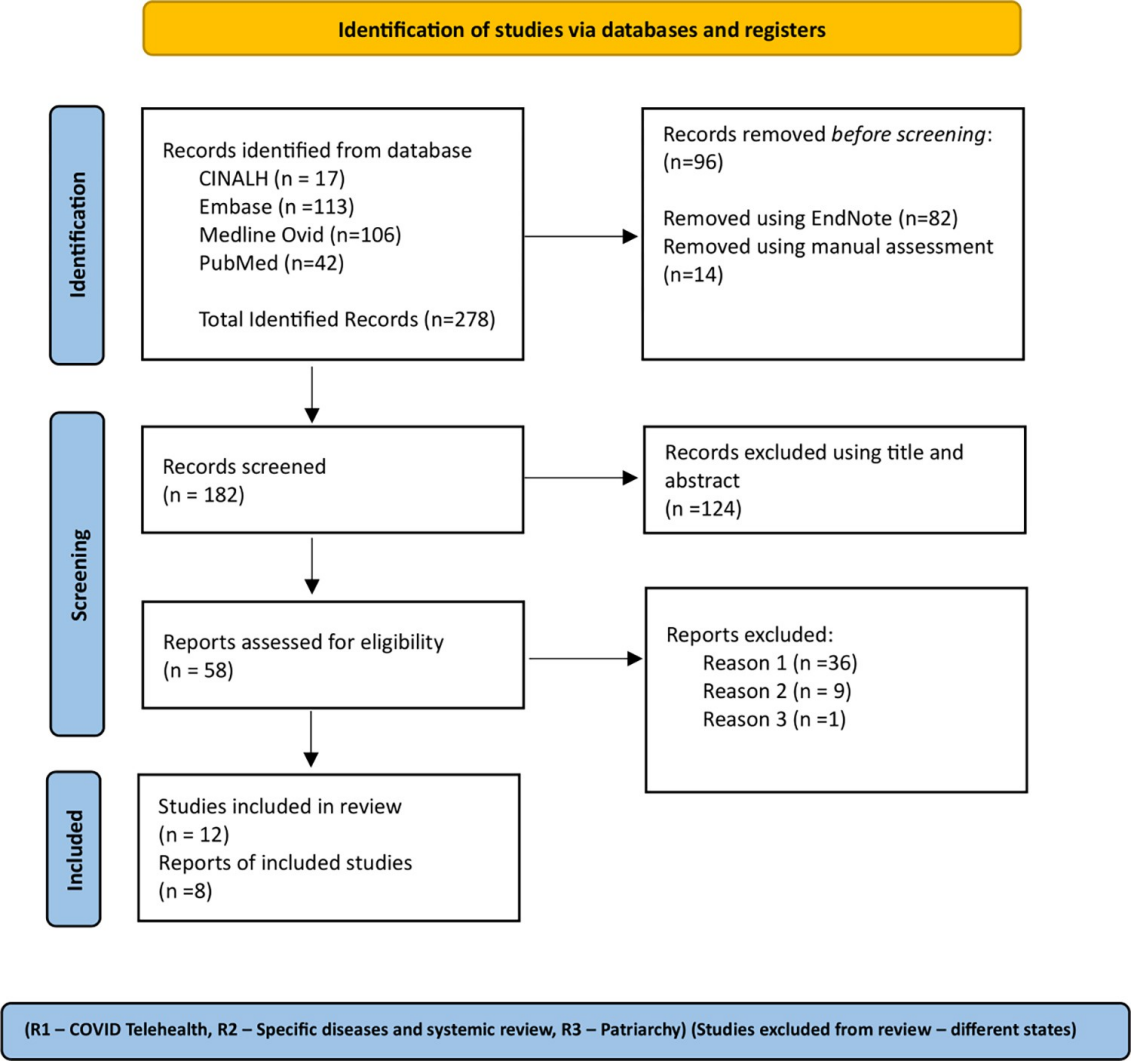
PRISMA Flow Diagram for Database Search of Studies on mHealth Interventions for Maternal and Child Health in BIMARU States of India (Page and colleagues). **(A)** The total number of records identified through the initial database search was 278. **(B)** 182 records remain after removing duplicate articles using EndnoteX9 and manual elimination (EndnoteX9 = 82 and Manual = 14). **(C)** 124 records remain after screening based on title and abstract. **(D)** 58 full-text articles were assessed to determine eligibility based on inclusion and exclusion criteria. **(E)** After full-text review, 46 studies were excluded due to specific reasons, such as being limited by technology (COVID Telehealth, Specific Diseases and Systemic Review, Patriarchy). **(F)** Only 12 studies met the eligibility criteria after full-text reading, and 8 were included in the final review after removing 4 more studies (limited by technology).

The initial search yielded 278 entries when the outcomes from all 4 databases were combined: CINALH (*n* = 17), Embase (*n* = 113), Medline (*n* = 106), and PubMed (*n* = 42). Duplicate articles were excluded using EndnoteX9 (82) followed by manual screening (14) based on title and abstract, leaving 182 records. Subsequently, among the 124 records, only 12 met the inclusion criteria (Table 1). Finally, 4 more studies were excluded after full-text reading (limited by technology), and 8 studies were included in the final review (Fig 3).

### Characteristics of included studies

The included studies took place in Bihar (*n* = 5) [27–31], Madhya Pradesh and Rajasthan (*n* = 2) [32,33], and Bihar and Madhya Pradesh (*n* = 1) [34]. None of the studies conducted in Uttar Pradesh were qualified for inclusion in this review. In 7 studies, the target population was frontline workers and beneficiaries (pregnant women and women with newborn, infant, and children and up to the age of 5 years); however, one study targeted medical professionals (doctors, nurses, midwives, etc.) [31]. The included studies that analyse the effect of mHealth applications and services on target populations can be found in S1 Appendix.

The data of all the included research are listed in Tables 2 and 3. The quality of each study and results of evidence appraisal for included studies are listed in Table 4.

**Table 2:** Data from individual included studies.

Individual study and study location	Study set up (methodology/research method/research design/sample size/sampling used)	Approvals granted/ethical issue arises	Critical analysis	Study conducted (procedure/data collection/data analysis)
Carmichael and colleagues Location: Bihar, India	• **Methodology**: RCT • **Research method**: Mixed method • **Research design**: Casual research, before and after with control group • **Sample selection**: stratified cluster Randomisation • **Sample size** 1. Control group: 134 AWW, 122 ASHA, and 809 maternal respondents 2. Intervention group: 154 AWW, 163 ASHA, and 744 maternal respondents	Institutional Review Board of the Public Health Foundation of India, New Delhi, and from the Health Ministry’s Screening Committee (approved on August 18, 2011);	Critical Appraisal Skills Program (**CASP**) tool for randomised controlled trial • Section A (validity of basic study design) = 3/3 • Section B (methodology assessment) = 2/3 • Section C (Results) = 2/3 • Section D (Result transferability) = 1/2 Score on appraisal: 8/11 Included	**Data collection:** Interviews at baseline and post-intervention Quantitative data (survey) **Data analysis:** Bivariate and difference-in-difference analyses, *p*-value calculated **Time horizon:** 2 years Baseline: May–July 2012 Post implementation: July–August 2014
Kaphle and colleagues Location: Bihar, India	• **Methodology:** Field observational • **Research method**: Mixed method • **Research design**: Descriptive, longitudinal • **Sample selection**: Block selection: Randomisation Further selection: Convenience sampling • **Sample size**: Intervention (*n* = 15), control group (*n* = 3)	**Respondents:** Consents Did not require any approvals from the Internal Review Board (IRB)	Critical Appraisal Framework for Original Research (CAFFOR) **Score on appraisal:** 6/7 Included	**Data collection**: • Interview (to assess the literary) • Formative study (observation of frontline workers during home visits after implementation of mHealth) **Data analysis**: Descriptive statistics, calculation of confidence interval, *p*-value, mean value, and standard deviation **Time horizon**: not mentioned Data was collected on 30th, 60th, and 90th day
LeFevre and colleagues Location: Madhya Pradesh and Rajasthan	• **Methodology**: iRCT (i = individual) • **Research method**: Mixed method • **Research design**: Casual relationship, Before and after with control group • **Sampling**: Randomisation (stratifications of important parameter) • Distribution in control and intervention group: Randomisation (using Stata) • Target Sample size: 3,200 (1,750 in each arm) • Response rate: 20%	Johns Hopkins School of Public Health Institutional Review Board in Baltimore, MD, USA, Sigma in Delhi India, and the University of Cape Town in South Africa	Critical Appraisal Skills Program (CASP) tool for randomised controlled trial • Section A (validity of basic study design) = 2/3 • Section B (methodology assessment) = 2/3 • Section C (Results) = 2/3 • Section D (Result transferability) = 2/2 Score on appraisal: Level of evidence: 9/11	**Data collection**: In-depth interview and Quantitative data collection and analysis **Data analysis**: • Statistical analysis, CI, contingency tables, and chi-square test • Economical evaluation: Cost-effectiveness analysis (calculation of DALY and ICER) Sensitivity and probability analysis Phone Survey analysis Time horizon: 2018–2019
Negandhi and colleagues Location: Bihar, India	• **Methodology**: Observational • **Research method**: Qualitative • **Research design**: Exploratory, case-study • **Sampling**: Convenience sampling of main participants • **Sample size**: Not mentioned	Written Consent from respondents Part of the INAP project, which was approved by the Institutional Ethics Committee of Indian Institute of Public Health Delhi	Critical Appraisal Skills Program (CASP) tool for Qualitative study • Section A (validity of basic study design) = 4/6 • Section B (Results) = 2/3 • Section C (Result transferability) = 1/1 **Score on appraisal**: 7/10 Included	**Data collection:** In-depth interview (face to face) **Data analysis:** Detailed manual analysis at emerging theme **Time horizon:** 2014–2015 (1 year)
Nimmagadda and colleagues Location: Bihar and Madhya Pradesh (MP), India	• **Methodology:** Quasi-experimental (controlled design), no blinding • **Research method**: Mixed method • **Research design**: Descriptive, repeated cross-sectional • **Sampling**: 1. Purposive sampling of state 2. PSM based sampling of village 3. Division in intervention and control group: Randomisation • Sample size: MP (*n* = 420), Bihar (*n* = 432)	Committee for the Protection of Human Subjects at the University of California at Berkeley, and the Suraksha Independent Ethics Committee in India. Informed consent as per the Institutional Review Board approved protocol.	Critical appraisal tool used: Joanna Briggs Institute (JBI) for Quasi-experimental study **Score on appraisal:** 8/9 Included Cause and effect’ are well elaborated, control group is present. Statistical analysis is performed, majority of sampling is randomisation, and statistical analysis is performed.	**Data collection**: Structured interview and survey questioner (pre- and post-intervention) **Tool**: android tablet based SurveyCTOTM platform **Data analysis**: Adjustment of data at baseline Data analysis done in STATA **Time horizon**: 12 months (baseline survey conducted in July–August 2017)
Usmanova and colleagues Location: Madhya Pradesh and Rajasthan, India	• **Methodology**: Observational • **Research method**: Qualitative • **Research design**: Exploratory, case-study • **Sampling**: Purposive sampling • **Sample size**: 12 facilities (6/39 in MP and 6/42 in Rajasthan) (3 low and 3 high utilizers) • **Response rate** = 91.6%	Written informed consent from respondents Oral recorded consent by respondents for telephonic interview Institutional Review Board (IRB) (IRB no. 10028) of the Johns Hopkins Bloomberg School of Public Health in the United States and the Sigma IRB (IRB no. 10065/IRB/19–20) in New Delhi, India. Permission for conducting the study was taken from both state government	Critical Appraisal Skills Program (**CASP**) tool for Qualitative study • Section A (validity of basic study design) = 4/6 • Section B (Results) = 2/3 • Section C (Result transferability) = 1/1 **Score on appraisal**: 9/10 Included	**Data collection**: In-depth interview (face to face) Lockdown on 24th March 2020 (COVID-19), 17 interview were conducted telephonic **Data analysis**: • Qualitative content analysis approach, TAM-3 model (data analysis software) **Time horizon**: 1 Year (July 2019–June 2019)
Ward and colleagues (2021) Location: Bihar, India	• **Methodology**: Observational (survey) • **Research method**: Mixed method • **Research design**: Descriptive, multiple cross sectional • **Sampling**: 1. Randomisation for selection of block 2. Purposive sampling of sample (women exposed to GSP and IPC) • **Sample size**: GupShup Potli (GSP) (*n* = 2,608) IPC tool (IPC) (*n* = 2,002)	Institutional Review Board of the Public Health Foundation of India, and from the Health Ministry’s Screening Committee on August 18, 2011. Ethical approval for data analyses done at Stanford University was received from the Stanford Institutional Review Board on 19 December 2016, protocol ID 39719.	Critical Appraisal Framework for Original Research (CAFFOR) **Score on appraisal**: 6/7 Included	**Data collection**: • Interviews and questioners, scripted for Computer Administered Personal Interviews using tablet computers • Survey Data was collected (July–September 2016) **Data analysis**: Secondary data analysis of multiple cross-sectional survey, Statistical analysis (*p*-value, *t* test, and CI calculation) Time horizon: (July–September 2016)
Ward and colleagues (2020) Location: Bihar, India	• **Methodology**: Observational, survey • **Research method**: Mixed method • **Study design**: Descriptive, longitudinal • **Sampling**: Multistage sampling approach 1. Randomise selection of geographical blocks 2. Randomised selection of village 3. Purposive sampling of women (listing was conducted) • **Sample size**: 1. Round 2–5: Intervention group: 1446; Control group: 4463 2. Round 6–9: Intervention group: 1628; Control group: 4688	ClinicalTrials.gov number NCT02726230 Institutional Review Board of the Public Health Foundation of India, and from the Health Ministry’s Screening Committee on August 18, 2011. Ethical approval for data analyses done at Stanford University was received from the Stanford Institutional Review Board on December 19, 2016, protocol ID 39719.	Critical Appraisal Framework for Original Research (CAFFOR) **Score on appraisal**: 7/7 Included	Data collection: • 3 individual survey • Self-reporting Collected by independent team under strict quality control **Data analysis**: Statical analysis using STATA (calculation of *p*-value, odds ratio, CI, *t* test, and χ^2^ tests) **Time horizon**: Community-based Household survey: 2012–2017 Mathematic’s Annaya evaluation: January–April 2014 User and engagement study: October–December 2014

**Table 3:** Data extracted from included studies.

Individual study	Intervention	Population	Outcomes
**Carmichael and colleagues**	Care evaluation and educational mobile application: Information Communication Technology-Continuum of Care Service (**ICT-CCS**)	• FLWs (ASHA and AWW) • Beneficiaries	Evaluation of effect on • Effectiveness in coordination between AWW’s and ASHA • Job performance • Healthcare behaviour
**Kaphle and colleagues**	Data collection mobile application: **CommCare**	• FLWs (ASHA and AWW’s)	Evaluation of impact of mHealth • Quality of delivered care • Experience of FLWs • Factors affecting the adaptability of mHealth
**LeFevre and colleagues**	Automated message delivery and reminding service: **Kilkari** (largest mobile based messaging program in World)	Beneficiaries	Evaluate • Effectiveness and cost effectiveness of Kilkari application
**Negandhi and colleagues**	Computer tablet-based Mother and Child Tracking System (MCTS)	Medical professionals (doctors, nurses, midwifes, etc.)	Evaluate • Process of implementation and the faced barriers and challenges • Possibilities of sustainability of intervention
**Nimmagadda and colleagues**	• Integrated Child Development Scheme and Common Application Software (ICDS-CAS)	• FLWs • Beneficiaries	1. Evaluate a. Number of home visits by FLWs 2. Evaluate b. Impact on ICDS services, knowledge, and practices on target population
**Usmanova and colleagues**	Support management during intrapartum period: Alliance for Saving Mothers and Newborn (ASMAN)	Health service providers	Evaluate: Barriers and acceptability of the intervention
**Ward and colleagues (2021)**	Community-based intervention • GupShup Potli (GSP) • Audio recordings or interpersonal communication (IPC)	• FLWs • Beneficiaries	Evaluate: • Effect of mHealth on FLW to deliver care in low resource setting • Compare health related knowledge and behaviour relation to exposure to
**Ward and colleagues (2020)**	Card-based tool paired with audio of fictional character: **Mobile kunji** and Dr Anita	Childbearing women and FLWs	Evaluate health related • Knowledge • Behaviours and attitude of target population

Terms used in table above

Frontline workers (FLWs): AWW’s and ASHA.

Beneficiaries: Pregnant or childbearing women (antepartum), women who recently gave birth (postpartum), children with age up to 5 years.

Anganwadi Workers (AWWs).

Accredited Social Health Activists (ASHAs).

**Table 4:** Evidence appraisal for included studies.

Individual study	Key findings	Strengths	Limitations	Validity and transferability of the evidence
**Carmichael and colleagues**	• Significant improvement in FLWs behaviours in RMNCHN ○ Significant increase in the job confidence among ASHA ○ Enhance coordination between ASHA and AWS ↑ effectiveness of FLW 11% ↑ home visit in 3^rd^ Trimester 12% ↑ in home visit 1^st^ week postnatal ○ 12%, 13% and 21% point ↑ in breastfeeding, skin to skin care and complementary feeding respectively	• Stratified random assignment in intervention and control arm ○ balance the size of both arm ○ reduce variation and ↑ statistical power • External piloting prior to launching the survey • Pre-post intervention data collection provided enhance comparison of outcomes • Response rate by maternal household: Average 90% • Respond rate: 92% ASHA and 97% by AWW’s • Repeated cross-section design minimises selection bias	• Different cohort took part in pre and post intervention data collection as time horizon was 1 year (women gave birth in 12 months) • Lack of baseline data precluded attribution of the difference to impact of the intervention Study design does not differentiate the impact of *Annaya Program*, which might impact the outcome • Limited information collected for FLWs supervision • Self-reporting by the respondent might resulted in reporting bias	• Evidence from the study could be generalised in similar population, geographic location, and setting (after evaluation of the budget and support) • Findings from the study will make appropriate contribution in literatures and future designee and implementation of mHealth intervention
**Kaphle and colleagues**	• Higher score or **higher user** of the intervention (CommCare), provided significantly higher quality and care experience • Age affected CommCare user negatively (↑ age ↓ use of mHealth and ↓ in care quality) • Literacy affect the CommCare use negatively (↓ in literacy ↓ in use of mHealth)	• RCT study design with ideal setting which decreases selection bias • Results divided into multiple themes and rigorous analysis was performed • **Descriptive analysis of the study** ○ User type of CommCare +ve and significantly correlated: Coefficient 0.771 (p = 0.001) with 99% CI ○ Significant +ve corelation between measures of quality: Coefficient 0.787 (p<0.001) with 99% CI • High reporting quality of the article (enhance engagement and limits misleading results)	• Too small sample size which affected the evidence conclusiveness on the findings • Unequal distribution of participants in control and intervention group which affect the outcome and might indicate sampling bias Study was limited to data from field work observation • Presence of researchers during the FLWs home visit might affected the performance of the participant, leading to performance bias	• Findings of the study could be used in tailoring new mHealth application and making it more user-friendly • Generalisability of the study is limited due to small size • Transferability: Findings or result could be used to informed hypotheses for further studies and could contribute in mHealth literature
**LeFevre and colleagues**	• Multiple barriers were identified which creates critical challenge in providing maternal mobile services (loss of phone, unactive network, late evaluation of pregnancy, etc.) • Calculated the Cost-effectiveness of the mobile program ICER was calculated	• RCT design with mixed method of data collection shows high quality of evidence validity and limited selection bias • Rigorous analysis of data using statistical analysis and power calculations • Full health-economic analysis to compare cost vs. effect of the intervention Sensitivity analysis was performed to eliminate any systematic bias Study promoted new standard “of linking data on individual level exposure to health outcome”	• Large sample size due to high population coverage could result in differences in characteristics which might suggest selection bias • The method of self-reporting by the respondents in home survey might suggest reporting bias • No attention has been paid behaviour performance and technology relationship	• Generalisability is limited as extra effort was used to gather telephone number for the study, which is not possible in large scale intervention • Results of the study could help in evaluating errors of technology in further designing of similar intervention (maternal mobile messaging initiatives) • Results from economic analysis could help decision-maker select most cost-effective strategy or intervention
**Negandhi and colleagues**	• Evaluated the effectiveness and feasibility of mHealth intervention • mHealth strength healthcare profession ○ ↑ quality ○ Completeness ○ Timeliness of delivering critical care	• Research question and aim of the study was clear • Rigorous and systematic analysis of qualitative data • Intervention was designed according to feasibility of the FLWs • Qualitative data collection was appropriate design to collected data related to implementation and feasibility barriers	• Convenience sampling methods might result in selection bias Reporting quality of the study is considered low as results, and limitation of study is not included in report • Important parameters like sample size is not mentioned	• Generalisability is low due to restricted demographic variance • Transferability: Data could be used to eliminate the technological errors in mHealth application or software and design of new application • Results can contribute in mHealth related literature
**Nimmagadda and colleagues**	• Implementation of mHealth ○ Improve effectiveness and efficiency of FLWs (ASHA and AWW’s) ○ ↑ job motivation ○ Improved beneficiaries ○ Improve knowledge of beneficiaries and FLW ○ ↑ FLWs home visit and real time data entry	• Risk related to validity of the findings were reduced by pre-intervention balancing • Repeated cross-sections increase the validity of study by reducing reporting bias • Blinding of beneficiaries to their intervention status • Mixed-method design build confidence in the study findings • Appropriate study design as gold standard RCT was not possible due to pre-determined program assignment	• Observational study design ○ Selection bias due to observative design ○ Measurement bias as outcomes were measured by interview • Decreased external validity due to sampling methods • High probability of presence of residual and unobserved confounding.	• Generalisability is limited due to study design • Finding will contribute evidence in evaluating the role of mHealth in increasing efficiency and effectiveness of healthcare workers
**Usmanova and colleagues**	• mHealth enhance the real-time data entry and eliminate human error • Technological and clinical training is essential to eliminate barrier in using mHealth • Improve quality of care and hence improve productivity of staff Barriers in use of mHealth: Lack of system flexibility Most common challenge is sustainability issue post pilot implementation of mHealth	• High response rate of 91.6% • Rigorous data analysis using software (TAM-3) and codebook was created with the help of team members • Response bias was limited by eliminating team members presence during interviews • Reliability of all codes were more than equal to 85% • All level of healthcare professional were involved in study enhance the generalisability of the result	• Selection bias: Purposive selection of higher and lower utilizers of the mHealth application • One-third of the interviews conducted remotely due to COVID-19, as it complicated rapport-building and might adversely affected data validity • “Jhpiego affiliation of the study” might resulted in response bias (respondent might gave socially desirable answers) • Study analysed perception rather than actual behaviour	• Transferability: structural and technological barriers could be used in planning similar mHealth intervention
**Ward and colleagues (2021)**	• Significant improvement in knowledge and health-related behaviours were observed in intervention group compared to control group	• Secondary data analysis using multiple cross-sectional survey (multiple cross-sections decrease selection bias as same candidate is not selected again) • Interview and questionnaires were scripted for computer administrated personal interview • Statistical analysis was performed using STATA to enhance the validity of findings • Odd ratio with respective 95% CI was reported for each indicator which shows high external validity of study	• Self-reported health-related behaviour might indicate reporting bias • Statistical analysis of data enhances the validity of findings • Due to COVID-19 lockdown 17 interviews conducted on phone which affect the data validity due to change in mode	• Generalisability is limited because intensive support and facilities were provided to the FLWs by BBC Media Action during the intervention • Scale of implementation (8 district and training of over 110 000 FLWs) and rigorous evaluation of multiple databased in the study can contribute unique findings in mHealth literature
**Ward and colleagues (2020)**	• Significant improvement in RMNCH related knowledge and health-related behaviour among target population (pregnant women) ○ Birth Preparedness and antenatal care practice ○ Postnatal behaviour (breast feeding and complementary feeding) • Significant impact on front-line workers self-efficiency and trust of their beneficiaries	• Sample selection by randomisation decreases selection bias • Rigorous evaluation across multiple data provided unique contribution Statistical analysis using STATA (power calculation like CI, *p*-value, etc.) which provided empirical value for evaluation • Three independent surveys • Appropriate sample size	• Self-reporting methods might affect the quality because of social desirability and response bias • Inequality in the size of control vs. intervention group might affected the outcome • Selection bias as FLW chose their beneficiaries	• Generalisability is limited because the study got intensive support from external sources (sustainability and scalability could be result of support) • Data and results could be used to perform unique contribution to literature on mHealth • Data could also be used to perform health technology assessment to find cost-effectiveness of the intervention, prior to implementation on a larger scale

### Appraisal of methodological quality

A critical evaluation study is considered a crucial step since it provides valuable details regarding research and reporting quality [35,36]. As a result, the quality of included studies is assessed using various critical appraisal tools based on the study approach shown in Table 2, and the individual study critical appraisal is attached in S1 Appendix. Whereas the Critical Appraisal Framework for Original Research (CAFFOR) is used to assess the strengths and limitations of all included studies.

#### Reporting and methodological quality of included studies

The reporting quality of the included articles is considered standard as the authors used standard methods and addressed important parameters like target population, intervention, location, research method, objectives, and increased readers’ transparency, understandability, reproducibility of its methods, and cross-examination of the results [37].

The majority of studies was mixed method designs and used in-depth interviews and surveys to evaluate the outcomes of the intervention. This data collection method was considered a justifiable option for included studies because quantitative data can provide analytical data and qualitative data can address social phenomena; a mixed-method design can provide detailed picture data by putting “words behind the numbers” [38]. Additionally, it can help in tailoring more efficient interventions and developing implementation strategies [39].

In contrast, sampling was done in stages, beginning with the selection of the state, block, district, and village, and then moving on to the sample population. Apart from 2 included studies [27,32], participants were selected by non-probability sampling as listed in Table 2, which raises questions about the external validity of these studies. However, using randomisation for the initial selection of the sample, prior to finalising the sampling, increased the internal validity of the studies by reducing selection bias [40]. Although the use of non-probability sampling at any stage implies selection bias, the standardised probability method is not always a viable option due to its rigidity [29,41,42]. For example, in the study by Ward and colleagues, researchers used randomisation to approach the real experimental design but later had to manually alter the sample due to a failed attempt brought on by participant error [29].

Selected studies showed meticulous analyses of the collected qualitative and quantitative data to boost accuracy by employing various methodologies. Interviews were manually analysed, transcribed, and translated by multiple private research experts, and software like STATA was used to minimise human error [31,33,43].

Nonetheless, one significant limitation inferred from the studies is the collection of self-reported data from participants [27–29,32], which is often argued to be “unreliable and threatened by self-reporting bias,” one of the common biases that affects research validity [44]. However, if the data are carefully collected and used, such as through pre-implementation instrument validation, prefacing and question design, selection of an acceptable recall time, and others, self-reporting can provide a wide range of information [44,45].

Another disadvantage is that almost all trials were deliberately tailored and monitored, which further questions the generalisability of the outcome, as extra support may not be feasible in a general context. For example, in the study by Lefevre and colleagues, researchers put extra effort into providing the correct phone numbers for the participants, which is not feasible in the usual setting of the Maternal and Child Health Tracking System (MCTS) [32]. Moreover, direct assessment of the performance and abilities of FLWs was another important component in the included studies, as the conscious awareness of being monitored enhanced the participants’ behaviours and performance according to the “Hawthorne effect” (sense of being monitored) [46]. Further details, including the key findings, limitations, strengths, and validity of each included study, are provided in Table 4.

### Evidence synthesis

The studies included in this literature review collected multiple pieces of evidence across multiple domains, and a thematic evidence synthesis was conducted using these data and merging similar observations. This synthesis of evidence resulted in the identification of 4 main themes along with multiple subthemes.

#### Theme 1: Effectiveness of the use of mHealth

According to the included literature, mHealth had a different impact on each target audience when compared with traditional systems. As a result, the theme is further divided into subgroups based on how mHealth has affected each category.

***Frontline health workers*.** The reviewed studies showed mHealth positively influences FLWs’ ability to deliver quality care to beneficiaries by improving FLWs’ efficacy and efficiency and raising their confidence (*P* < 0.05) in a number of abilities, including visit planning, and maintaining records of maternal and child difficulties [27,33,34]. According to Carmichael and colleagues, FLWs exposed to the mHealth intervention needed less training and communication skills to effectively interact with beneficiaries [27]. Additionally, they build the capability of monitoring and updating pertinent data, identifying already provided services against those that are still needed [27]. Moreover, Usmanova and colleagues claim that mHealth improves the FLWs ability to conduct in-depth histories and reduce variation as the application requires methodological entry of data in a “step-by-step” manner [33].

*Healthcare professionals*. Although only a few studies address the effectiveness of mHealth applications on healthcare professionals like medical officers, staff nurses, auxiliary nurse midwives (ANMs), labour room supervisors, etc. [33,34], they do not engage healthcare professionals directly beyond the initial evaluation process. However, positive impacts were observed in these studies in terms of control, coordination, and supervision. The study done by using the ASSMAN application for mHealth, “facilitates timely and accurate clinical decision-making by providers at project sites,” and the management alert notification feature of the application empowered the nurses to improve their job performance by handling complex cases [33,34].

*Beneficiaries*. Four studies were included in the review to determine whether use of an mHealth intervention tool on beneficiaries, whether used by FLWs or beneficiaries themselves, would result in improved health outcomes [27–29,34]. The main parameters assessed in terms of effectiveness are antenatal care, consumption of supplementary medication, birth, or delivery preparedness, early and exclusive breastfeeding, skin-to-skin care, and complementary feeding. Studies reported that women exposed to mHealth were 3 times more likely to take measures for delivery preparedness such as saving money, gathering important contact information, and identifying transportation modes [28]. The intervention group also showed increased frequency in pregnancy registration (80.4% versus 75.3%, *p* < 0.001) and exclusively breastfed their new born until the age of 6 months (58% versus 42.5%, *p* < 0.01) [28].

Furthermore, Nimmagadda and colleagues claimed that mHealth had a positive impact on the exposed group’s knowledge and health behaviours in all indicators of prenatal and postnatal care [34]. The studies by Ward and colleagues (2021) and Ward and colleagues (2020) provided quantitative data for evaluation of the effectiveness of mHealth on beneficiaries (odd ratio (OR), *p*-value, and confidence interval (CI) summarised in Table 5 [28,29].

**Table 5:** Data of respondents exposed to mHealth.

Stages	Odd ratio	Confidence interval (CI)	*P*-value
**Iron-folic acid (IFA) tablets**	1.5 2.3	95% CI = 1.1–2.2 95% CI = 1.8–3.1	-
**Pregnancy registration**	1.64	95% CI = 1.37–1.98	<0.001
**Delivery preparedness**	2.8 1.3	95% CI = 1.9–4.2 95% CI = 1.0–1.7	0.025 0.03
**Early breast feeding**	1.64	95% CI = 1.5–1.78	<0.001
**Exclusive breast feeding**	1.46 1.8	95% CI = 1.33–1.62 95% CI = 1.3–2.7	<0.001
**Complementary breast feeding**	1.9 1.6	95% CI = 1.0–3.5 95% CI = 1.2–2.2	<0.001

Note: Two values in few cells are from 2 different studies (Ward and colleagues, 2020) and (Ward and colleagues, 2021).

Additionally, Carmichael and colleagues found a 13% increase in skin-to-skin care, a 12% increase in early breast feeding immediately after delivery, a 21% increase in complementary feeding, and a significantly higher percentage of exposed participants (54.4% versus 37%) sharing the gained knowledge with others, particularly their family [27,28].

**Table 5:** Data of respondents exposed to mHealth quantitative data showing effectiveness of on beneficiaries (Ward et al., 2020) and (Ward et al., 2021)

#### Theme 2: Feasibility

Almost all of the included studies in the review assessed the sample population’s feasibility to use mHealth. Studies tried to understand the complexities in the design as well as the challenges faced by the users in terms of acceptability and adoptability of the intervention. The studies covered a range of different parameters, which are subdivided into technical, economical, legal, and operational feasibility.

*Technical feasibility*. The studies in the review covered a range of maternal and child health domains and mHealth applications listed in Table 3 under the intervention category. All studies considered training as one of the most beneficial and important parts of mHealth application feasibility. According to Usmanova and colleagues, adequate training provided the knowledge and skills required to use mHealth and sped up the work compared to handwritten records [33]. Training also improves the management skills of the users and makes record keeping and monitoring easier by, “enhancing perceived ease of use” [33]. The benefits of mHealth have been documented in peer-reviewed studies, including an increase in home visits and follow-up (antenatal visit post implementation, *p* = 0.05) [27], an improvement in patient monitoring, improved decision-making, an increase in self-efficacy and empowerment, and an increase in level of confidence [33].

On the contrary, some studies reported technological challenges hindering the proper utilisation of mHealth like internet connection issues, data loss, occasional bugs while doing offline data entry, database inflexibility, and difficulty in changing incorrect entries [33]. However, Carmichael and colleagues reported 85% of participants encountering no issues with mobile battery charging, and only 14% reencountered issues [27].

*Economic feasibility*. Only 2 studies examined the cost-effectiveness of an intervention for mobile health and claimed that as mHealth applications can be freely downloaded several times to any multimedia device, through the Google Play Store, they are both a cost-effective and “self-sunning” intervention [31,32]. Furthermore, there are no additional costs associated with the application’s upgrade [31]. However, LeFevre and colleagues have not provided any evidence regarding this parameter [32].

*Ethical feasibility*. Almost all the included studies provided strong evidence of ethical approval granted from legitimate sources (*Table 2*) and did not report any ethical issues raised during the time horizon of the conducted studies. Kaphle and colleagues’ research did not require any approval from the Internal Review Board (IRB), as their research did not involve patients or patient outcomes; however, written consent was obtained from the participants (ASHA) prior to the study [30].

*Operational feasibility*. One of the most promising features of mHealth, as demonstrated by the included studies, is real-time data collection, which allowed for immediate data uploading to a server and the generation of daily work plans. According to a few studies, this real-time dashboard and the availability of pertinent data in a central database effectively decreased the time between shifts (one task to another) and facilitated continuity of care [27,31]. Further, this central database of participant information can be used to communicate health-specific messages and reminders to beneficiaries’ mobile phones, facilitating health education and behaviour change [28,29,33]. These patient records might be used in an emergency in the absence of physicians to provide best available services to the patient, which improves accountability of nurse practitioners or emergency healthcare professionals [27].

Additionally, mHealth facilitated an automatic home visit schedule and a timely reminder for FLWs, which increased regular visit frequency (intervention group 72% versus the control group 60%, *P* < 0.01) [27,34]. During these home visits, FLWs spent an average of 17%, 39%, and 16% of their time delivering information by playing videos, audio material, and reading the information list, respectively [27]. Furthermore, additional mHealth functionality, such as the availability of multiple health-related videos and the capture of images for monitoring, aides diagnosis and treatment by evaluating clinical trends, assisting FLW in decision-making, planning courses of action planning, and providing an e-learning platform [31].

Usmanova and colleagues found that mHealth increased workload because employees had to perform duplicate entries (firstly in paper records and then on mobile application), and the researchers used a dummy server that was not connected to the server for the national MCTS (Mother and Child Tracking System) portal [33].

#### Theme 3: Barriers to acceptability

**Fig 4:**
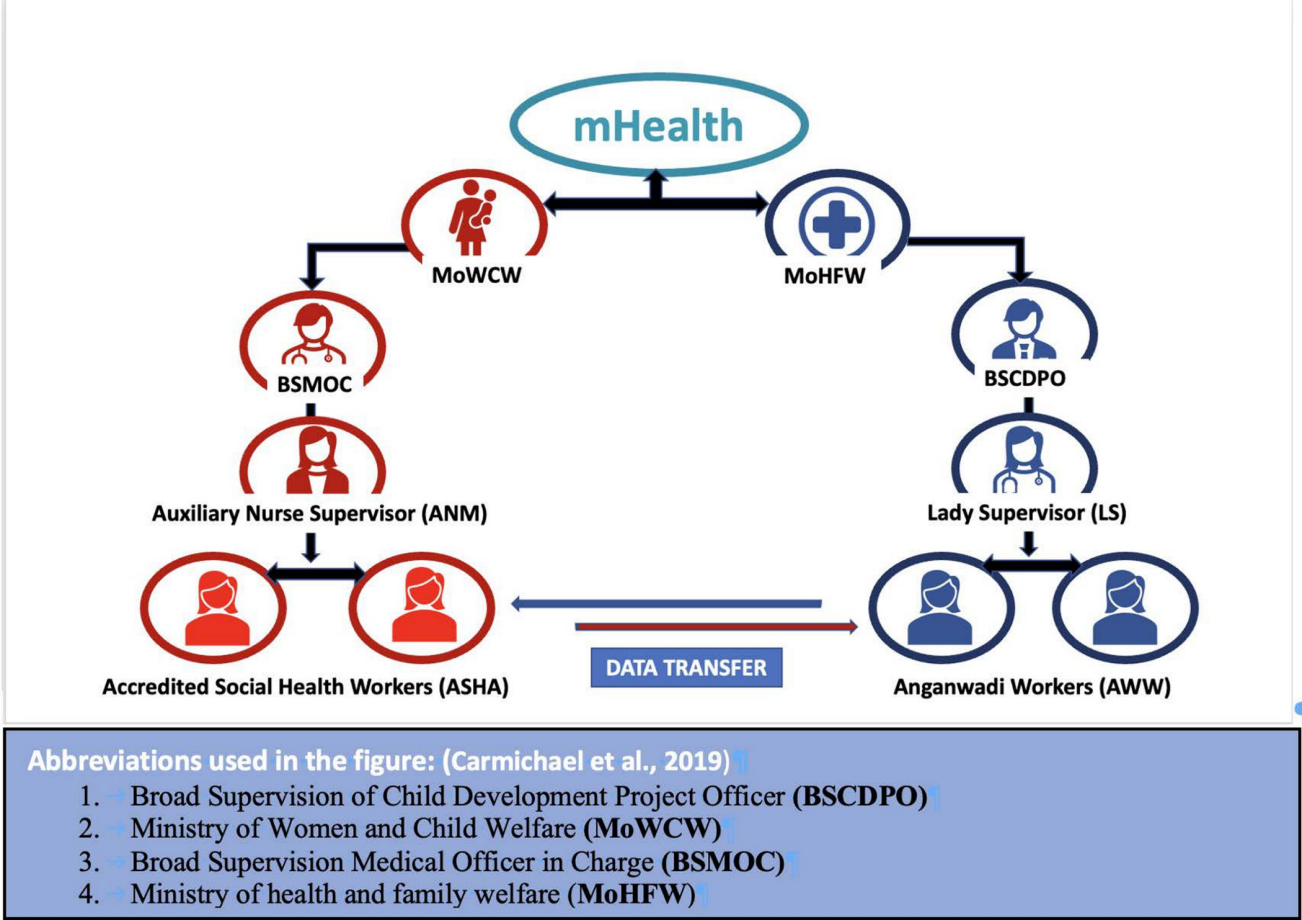
Relationship between ASHA and AWW in data transfer using mHealth. **(A)** Both ASHA and AWW share common objectives in promoting maternal and child health (data is used from Carmichael and colleagues to create this figure). **(B)** Ministry of Health and Family Welfare (MoHFW) manages ASHA (Accredited Social Health Activist) program. **(C)** Ministry of Women and Child Development (MoWCD) manages AWW (Anganwadi Worker) program. **(D)** mHealth platform facilitating data transfer between ASHA and AWW. **(E)** Blue colour arrow shows data transfer from AWW to ASHA and red arrow shows data transfer from ASHA to AWW.

This review identified multiple common challenges faced by the target population while using mHealth interventions and the inferred 4 main barriers can be addressed programmatically. First, there are technological barriers, such as issues with the internet, a lack of electricity to charge the tool, and device loss or breakdown [27,31,33]. According to Carmichael and colleagues, only 14% of users reported problems with the device [27]. Second, there is the cultural barrier as same phone is used by the whole family of the FLW due to socioeconomical and cultural barriers, which affect the process of delivering as well as receiving important messages [31].Third are structural barriers, such as challenges related to role shifting or coordination between 2 healthcare providers. A lack of coordination between ASHA and AWWs was identified, which showed a significant negative impact on mHealth acceptance as both categories shared the same primary targets (mothers and children) but were administered by different government ministries (Fig 4), hence creating a structural barrier [27].

Fourth was the social component, which includes things like age, education, and experience with technology. According to a study by Kaphle and colleagues, age has a negative impact on the use of mobile health tools: when “low and middle levels of literacy and education were combined into one variable,” they found that as FLW age increased, the likelihood that the FLW would use mobile health tools decreased (−0.105, *p* = 0.08 and 95% CI) [30]. They also discovered that illiteracy lowers the mHealth competence score (41% at the 95% CI), whereas prior experience with other similar technology raises the score, but user characteristics have no influence on mHealth application adoptability [31].

#### Theme 4: Communication and trust

Several studies have found that using mHealth can improve communication between frontline workers (ASHA and AWWs), between different levels of medical professionals (medical officers and nurses) and allow the supervisor to assess the FLWs performance [27,33,34]. Usmanova and colleagues argued mHealth would bridge the communication gap between nurses and medical officers and empower staff nurses by encouraging timely discussion to manage complex situations [33]. Carmichael and colleagues found that the experiment and control groups had similar experiences with supervision and received timely help in person and over the phone [27]. Moreover, mHealth increased coordination between ASHA and AWWs on home visits and enhanced their job confidence [27].

Furthermore, results showed a direct relationship between mHealth and trustworthiness, indicating that trust between beneficiaries and FLWs in the intervention group was generally stronger than in the control group (94.4% versus 86.5%, *p* < 0.001) [29].

## Discussion

### Principal findings

This systematic review evaluated the effectiveness, feasibility, and barriers related to acceptance of utilising mHealth technology among key stakeholders, i.e., FLWs, beneficiaries, and healthcare practitioners, in the BIMARU states of India. In this review, various types of mHealth platforms were considered with the goal of improving quality, equity, and service delivery efficiencies in healthcare, and significant improvements in all key indicators were found in the intervention group compared to the control group.

The analysis also demonstrated significant positive and promising results in terms of feasibility, effectiveness, and acceptability, as well as a change in behaviour and attitude in accordance with maternal and child healthcare, when mobile technology like mobile phones and tablets are used in the place of traditional methods. Labrique and colleagues argued mHealth is a “constituted health system strengthening technology,” and its advanced functions enabled the caregivers to track their patients, monitor their data remotely, and streamline referrals [13]. These features enhance their knowledge and skills and directly impact the quality of delivered care [47].

Likewise, Balakrishnan and colleagues stated that interventions that improve equitable access to healthcare, effective real-time data collection, and behaviour have the potential to strengthen the health system [48]. Similarly, a study conducted in South Africa also supported the use of mHealth by FLWs and CHWs (community health workers) as a job aid, which led to better health-seeking behaviour [49].

### Effectiveness of the use of mHealth

This review clearly indicated that mHealth shows favourable results in facilitating the effectiveness of FLWs in highly resource-constrained settings. mHealth improves FLWs behaviour towards delivering care by boosting their confidence and increasing their coordination and communication with other healthcare workers [27,33,34]. It also increases FLWs home visits, which enhance their knowledge and understanding of RMNCH behaviours and encourage beneficiaries’ adherence to recommendations and treatments [50,51]. Furthermore, improvements in HCW behaviour have the potential to reduce maternal and child mortality rates by improving care quality [52,53].

Reviewed studies showed a uniform agreement regarding the usefulness of mHealth for quality assurance among all healthcare professionals [33,34]. Usmanova and colleagues used the term “perceived ease of use,” meaning an easy-to-use platform that generates high-quality medical documents by reducing human error. Respondents reported that mHealth helped in improvement of knowledge, managerial skills, history taking, physical examination, and timely referrals [33]. Likewise, numerous studies also reported similar findings, like an improvement in the quality of the clinical database and the identification of signs and symptoms of complications such as stillbirth [54].

Only a few studies, however, rigorously evaluated the direct effectiveness of mHealth on mothers, and they were limited to birth preparation and postpartum care behaviours and attitudes [27–29,34]. Jennings and colleagues identified mHealth as a catalyst that increases the involvement of fathers and provides support to new mothers, which is considered an important norm by the WHO for improving prenatal and postnatal care [55,56].

### Feasibility

The emergence of new technologies such as mHealth is accompanied by multiple new norms, and establishing feasibility is considered a priority in such an evolution. The reviewed data suggests that collective acceptance among all stakeholders occurs when the integration is tailored strategically in accordance with the target population’s socioeconomic and demographic characteristics [57].

#### Technical feasibility

This review suggests that mHealth is a mature technology, and research supports its feasibility and significant rate of adoption in related contexts [56,58]. Numerous research claims that mHealth interventions are user-friendly, and this trait boosts the interventions’ usability and effectiveness [33]. Furthermore, data supports the fact that using wireless technologies through mHealth interventions can achieve modernization and streamlining goals in healthcare [59].

#### Economic feasibility

Every change comes with a cost and considering mHealth as a change intervention for strengthening the healthcare delivery system, multiple associated costs come into consideration, such as those associated with the technological infrastructure and implementation of the intervention. Given the low socioeconomic status of BIMARU states, the cost-effectiveness of the intervention plays a crucial role in assessing the economic feasibility of the intervention. Due to fewer studies regarding the economic evaluation of the mHealth intervention and limited data from included studies in this parameter, it is difficult to make a compelling argument for the cost-effectiveness of this intervention due to the absence of supporting evidence. However, Nimmagadda and colleagues discussed a few factors that highlight the cost-effectiveness of this intervention, and this evidence was also supported by a few other studies that state that involvement of multiple private and charitable stakeholders in the intervention can make mHealth interventions cost-effective [34,60–62].

#### Ethical feasibility

mHealth technologies are becoming ubiquitous, and the collection of patients’ data is expanding continuously. Hence, end-user protection and ethical issues are considered important deciding factors of this intervention’s feasibility and could play a valuable role in fabricating a more stable intervention or conducting health research [63]. In the year 2015, the WHO proposed 4 bioethical principles, namely autonomy, beneficence, non-maleficence, and justice, associated with transparency and confidentiality of data as the code of mHealth ethics to minimise the rise of ethical uncertainty and unregulated use of collected data [63,64]. None of the included studies provided any relevant evidence regarding upholding these principles [65]. However, the review concluded that no ethical issues arose during the research in any of the studies included.

#### Operational feasibility

There are multiple positive functions enumerated under the strength of the mHealth technology in the included studies, and these factors enhance the adoptability of mHealth [66,67]. Few respondents reported increased workload because of double data entry (one digital and the other on paper) [33]. Similarly, a maternity care study conducted in rural regions of Tanzania and Ghana suggested double documentation as a primary barrier to the adoption of mHealth technology [67].

Although there are constraints that limit mHealth potential, such as underutilised tools and India’s unstable healthcare system, evidence from existing literature and this review supports the claim that training is the most important asset for improving user operational feasibility and assisting in the management of behaviour change in healthcare professionals [7,27,29,31].

### Barriers to acceptability

The potential benefits and consistent growth of mHealth are supported by a wealth of research, yet numerous studies have revealed signs of resistance to its acceptance [68]. The researcher found a number of barriers, but the technological barrier was the most frequent one. Commonly reported issues like data uploading, system errors, poor output quality, and missing data sometimes discourage users from using mHealth [27,31,33] and cause disruption in the delivery of care [69,70]. The availability of 24-h technical support could impact the attitude of caregivers towards the acceptability of mHealth because of their round-the-clock services, and a lack of support might create difficulties in adaptability [71]. Another issue to consider is poor network and internet access, which may impair data entry quality and speed, reducing the tool’s utility [31,72,73].

Despite the increased use of mobile phones in LMICs, there is still a gender gap in mobile phone access, and women have limited accessibility to mobile devices because of cultural, social, and economic barriers [74]. According to a survey in India, approximately half of the population shares phones with their family members, including FLWs with phone ownership [31,74], which can violate data confidentiality, undermine bioethical principles, and violate the code of mHealth ethics [64].

Following the Alma Ata Declaration by the WHO in 1978, both categories of community health workers (CHW), ASHA and AWW, gained prominence in the healthcare field [1]. Furthermore, both ASHA and AWWA share a wide range of objectives and have to coordinate and shift roles to serve the same beneficiaries. However, it was observed that the primary structural impediment to the acceptance of mHealth was AWWA’s permanent employment and fixed incentives for the same duties as ASHA, as they were overseen by a different health administration structure. This structural difference was noted to be the main reason for conflicts of interest, creating a barrier to the acceptance of mHealth [27,75]. Although modifications to the mHealth programme could not eliminate these disparities [27].

Finally, moderating factors like age, literacy, and prior mobile technology use were found to have an impact on adoption intention in the included papers [30,31]. Similarly, evidence suggests that familiarity with mobile phones can boost confidence and usability, but limited understanding and experience with mobile phones causes confusion about usability and can be classified as an mHealth acceptance barrier [30,69,76].

Similarly, the age factor was also highlighted as negatively affecting mHealth’s acceptability, with younger users showing a more positive attitude toward embracing new technology. These barriers could be minimised by enhancing users’ talents through appropriate training [30,31,72]. Although some research has argued that the adoption of new technology is not affected by age disparities [77,78].

### Communication and trust

The primary function of mobile phones is communication; hence, mHealth depends on well-functioning communication infrastructure. Studies reflect that mHealth can aid in the development of FLW communication abilities, allowing them to communicate effectively with beneficiaries, which further improves their relationship with beneficiaries [27,33,34]. A similar conclusion of improvement in communication skills with the use of mHealth-based communication was reached by existing literature [28,55,79,80].

Moreover, mHealth technology empowers FLWs and beneficiaries with cutting-edge knowledge and communication skills in order to improve healthcare quality for people in difficult-to-reach regions. According to Ward and colleagues, FLWs use of mHealth for communication is more trusted by their beneficiaries [29]. Their beneficiaries adhered to healthy practises, recalled their messages, and discussed their relevant knowledge with others [29,81,82].

### Strengths

This review conducted a comprehensive, systematic literature search strategy based on the best available data to analyse the influence of utilising mHealth to strengthen maternal and child health in India. The study successfully captured different legitimate mHealth functions [19,20]. The methodology used in the narrative synthesis looked at in-depth functions of mHealth by targeting both healthcare providers and beneficiaries to help synthesise the effect of mHealth’s impact on all primary stakeholders, a technique that made it possible to evaluate the value of various mHealth services [19,20]. Although the number of included articles was lower due to the lack of research availability in these BIMARU states, this review contributes evidence that might be used in the management and implementation of a successful mHealth intervention targeting maternal and child health in a comparable context.

Additionally, researchers used repeated cross-sectional analysis because the cohorts at baseline and post-intervention analysis were significantly different because the baseline cohort had already given birth within the time horizon [27,29,34]. This strategy minimised selection bias by improving the representativeness of the participants [27].

Conversely, researchers used observational designs as they provide behaviour and attitude-related information for multiple studies; however, although the inherited systematic limitations of observational studies decrease the external and internal validity [41]. These inherited limitations are minimised by avoiding investigators’ presence during data collection, as their presence might affect participants’ behaviour, leading to response/participant bias [41]. However, data reliability remains a concern because participant and researcher bias may still exist because both responses and observations are subjective [42].

### Limitations

Despite the fact that this literature review contributes to the existing literature on mHealth, there are limitations that must be acknowledged and need to be addressed in future research. This analysis is limited to articles from 4 electronic databases published in English since 2012, and no further communication with the authors was undertaken in order to get additional material or to corroborate our thematic analysis. However, since the first mHealth tool was introduced in India in the year 2010 and initial tool piloting was performed in the year 2012, it is appropriate to limit the search to that year [83]. Likewise, English-language is used in approximately 86% of published articles worldwide and is considered the preferred language in medical research publications; hence, using English-language articles does not appear to have led to “English-language bias,” [84,85].

The scope of this review domain is limited to the BIMARU states of India with an unequal distribution of studies among these included states, which might indicate observation bias, though this is somewhat mitigated by the striking geographic, population, socioeconomic, and demographic similarities between BIMARU states. However, the limited scope of our review might affect the generalizability of the results to different settings [86].

## Conclusions

The mHealth intervention is part of a comprehensive initiative in India that gives the government, healthcare professionals, and policymakers the chance to enhance the current healthcare system. Therefore, a detailed analysis of the components that contribute to the development and implementation of a successful strategy is required. The BIMARU states could be used as an example because they show India’s soft underbelly (weakest part) when social development is considered. As a result, India’s progress is closely correlated with the advancement of any significant development aspects in these BIMARU state affairs. This review highlights how various mHealth technologies can be used to improve maternal and child healthcare in low-resource settings and lays out the structure of an mHealth intervention for India’s BIMARU states.

This review also offers data showing that mHealth reduces the main challenges faced by people living in low-resource settings when trying to access healthcare facilities, such as accessibility, affordability, and availability. Additionally, it also highlighted numerous issues, including barriers to acceptance of this mHealth, such as technological, cultural, social, and structural issues, as well as ethical concerns related to patient autonomy and data protection.

Overall, mHealth interventions can be said to strengthen the healthcare system because of their impact on the quality, efficacy, and equity of delivered care services, as well as the effectiveness and feasibility among care providers and recipients, but further research would be necessary to draw any strong conclusions. Likewise, more studies utilising rigorous methodologies, such as true RCTs, are required to provide reliable and accurate information to back up the conclusions of this review.

## Supporting information

S1 PRISMA Checklist Preferred Reporting Items for Systematic reviews and Meta-Analyses extension for Scoping Reviews (PRISMA-ScR) Checklist. (DOCX)

S1 Appendix (Supporting information). (DOCX)

S2 Appendix (Process of selection and elimination of articles). (DOCX)

S1 Text Search string of each database. (DOCX)
