# Supplementary material for: Use of mHealth in promoting maternal and child health in “BIMARU” states of India “A health system strengthening strategy”: Systematic literature review
Source: PLOS Digit Health. 2024 Feb 2;3(2):e0000403. doi: 10.1371/journal.pdig.0000403 (PMC10836675; doi:10.1371/journal.pdig.0000403)
Supplement: S1 Appendix — (DOCX) [file pdig.0000403.s002.docx]

**S1 Appendix:**

***Main causes of maternal death and key interventions*** (*Source: Series Papers Maternal Health 1 and 6*)

| MeSH Terms Use in Electronic Database Search | |
| --- | --- |
| Category | **MeSH Term** |
| Population | ‘Community health worker’, ‘care givers’, ‘health personnel’, healthcare professional’, ‘Frontline worker’, ‘health service’, ‘ASHA’, ‘AWW’, ‘maternal health service’, ‘child health service’, ‘new-born healthcare’, ‘mothers health care’, ‘FLW’, ‘CHW’ |
| Intervention | ‘mHealth’, ‘mobile health’, ‘ehealth’, ‘m-health’, ‘mobile app’, ‘smart phone’ |
| Outcomes | ‘effectiveness’, ‘effect’, ‘adaptation’, ‘adoption’, ‘acceptability’, ‘feasibility’, ‘behaviour’, ‘attitude’, ‘Communication’, ‘acceptance’, ‘barriers’ |
| Place | ‘BIMARU’, ‘Bihar’, ‘Uttar Pradesh’, ‘UP’, ‘Madhya Pardesh’, ‘MP’, ‘Rajasthan’, ‘India’ |

| PICO Format of Research Question | |
| --- | --- |
| Population (P) | Pregnant women, mothers, children’s, FLW, and healthcare professionals |
| Intervention (I) | mHealth or Mobile Health (different applications) |
| Comparison (C) | Traditional paper base or non-technological method |
| Outcomes (O) | - Effectiveness of mHealth on target population - Feasibility of mHealth intervention - Barriers and challenges in accepting mHealth |

| Details of all included studies | | | |
| --- | --- | --- | --- |
| Sr No. | **Study** | **State** | **Title** |
| 1. | Carmichael et al., 2019 | Bihar | Use of mobile technology by frontline health workers to promote reproductive, maternal, new-born and child health and nutrition: a cluster randomized controlled Trial in Bihar, India |
| 2. | Kaphle et al., 2015 | Bihar | Adoption and Usage of mHealth Technology on Quality and Experience of Care Provided by Frontline Workers: Observations from Rural India |
| 3. | LeFevre et al., 2019 | Madhya Pradesh & Rajasthan | Are stage-based health information messages effective and good value for money in improving maternal new-born and child health outcomes in India? Protocol for an individually randomized controlled trial |
| 4. | Negandhi et al., 2016 | Bihar | Computer tablet-based health technology for strengthening maternal and child tracking in Bihar |
| 5. | Nimmagadda et al., 2019 | Bihar and Madhya Pradesh | Effects of an mHealth intervention for community health workers on maternal and child nutrition and health service delivery in India: protocol for a quasi-experimental mixed-methods evaluation |
| 6. | Usmanova et al., 2020 | Rajasthan and Madhya Pradesh | Acceptability and Barriers to Use of the ASMAN Provider-Facing Electronic Platform for Peripartum Care in Public Facilities in Madhya Pradesh and Rajasthan, India: A Qualitative Study Using the Technology Acceptance Model-3 |
| 7. | Ward et al., 2021 | Bihar | Implementing health communication tools at scale: mobile audio messaging and paper-based job aids for front-line workers providing community health education to mothers in Bihar, India |
| 8. | Ward et al., 2020 | Bihar | Impact of mHealth interventions for reproductive, maternal, new-born and child health and nutrition at scale: BBC Media Action and the Ananya program in Bihar, India |

**Identification of studies via databases and registers**

Records removed *before screening*: (n=182)

Removed using EndNote (n=82)

Removed using (n=14)

Records identified from database

CINALH (n = 17)

Embase (n =113)

Medline Ovid (n=106)

PubMed (n=42)

Total Identified Records (n=278)

**Identification**

Records screened

(n = 186)

Records excluded using title and abstract

(n =124)

**Screening**

Reports excluded:

Reason 1 (n =36)

Reason 2 (n = 9)

Reason 3 (n =1)

etc.

Reports assessed for eligibility

(n = 62)

Studies included in review

(n = 16)

Reports of included studies

(n =8)

**Included**

**(R1 – COVID Telehealth, R2 – Specific diseases and systemic review, R3 – Patriarchy) (Studies excluded from review – different states)**

**Figure 3**: PRISMA flow diagram for database search of studies on mHealth interventions for maternal and child health in BIMARU states of India (Page et al., 2020)

| *Critical Appraisal Skills Programme*  Critical Appraisal Skills Programme for Randomised Control Trial Study  RANDOMISED CONTROL TRIAL | | |
| --- | --- | --- |
| Individual study | **Carmichael et al., 2019** | **LeFevre et al., 2019** |
| Section A: Is the basic study design valid for a randomised controlled trial? | | |
| 1: Did the study address a clearly focused research question? | **Yes** | **Yes** |
| 2: Was the assignment of participants to interventions randomised? | **Yes** | **Yes** |
| 3: Were all participants who entered the study accounted for at its conclusion? | **Yes** | **No** |
| Section B: Was the study methodologically sound? | | |
| 4: Blinding | **Can’t tell** | **No** |
| 5: Were the study groups similar at the start of the randomised controlled trial? | **Yes** | **Yes** |
| 6: Apart from the experimental intervention, did each study group receive the same level of care (that is, were they treated equally)? | **Yes** | **Yes** |
| Section C: What are the results? | | |
| 7: Were the effects of intervention reported comprehensively? | **Yes** | **Yes** |
| 8: Was the precision of the estimate of the intervention or treatment effect reported? | **No** | **Yes** |
| 9: Do the benefits of the experimental intervention outweigh the harms and costs? | **No** | **Yes** |
| Section D: Will the results help locally? | | |
| 10: Can the results be applied to your local population/in your context? | **Yes** | **Yes** |
| 11: Would the experimental intervention provide greater value to the people in your care than any of the existing interventions? | **Yes** | **Yes** |
| TOTAL: 11 | **8** | **9** |

| Critical Appraisal Skills Programme Qualitative Study | | |
| --- | --- | --- |
| Individual study | **Negandhi et al., 2016** | **Usmanova et al., 2020** |
| Section A: Are the results valid? | | |
| 1: Was there a clear statement of the aims of the research? | **Yes** | **Yes** |
| 2: Is a qualitative methodology appropriate? | **Yes** | **Yes** |
| 3: Was the research design appropriate to address the aims of the research? | **Can’t Tell** | **Yes** |
| 4: Was the recruitment strategy appropriate to the aims of the research? | **Yes** | **Yes** |
| 5: Was the data collected in a way that addressed the research issue? | **Yes** | **Yes** |
| Section B: What are the results? | | |
| 6: Has the relationship between research and participants been adequately considered? | **Can’t Tell** | **Can’t Tell** |
| 7: Have Ethical issues been taken in consideration? | **Yes** | **Yes** |
| 8: Was the data analysis sufficiently rigorous? | **No** | **Yes** |
| 9: Is there a clear statement of findings? | **Yes** | **Yes** |
| Section C: Will the results help locally? | | |
| 10: Was result valuable for your research? | **Yes** | **Yes** |
| TOTAL | **7** | **9** |

| JBI Critical Appraisal Checklist for Quasi-Experimental | |
| --- | --- |
| Individual study | **Nimmagadda et al., 2019** |
| 1: Clarity of cause and effect | **Yes** |
| 2: Similarity between participants included in comparisons | **Yes** |
| 3: Similar treatment/care among participants | **Yes** |
| 4: Control group | **Yes** |
| 5: Multiple measurements of the outcome both pre and post the intervention | **Yes** |
| 6: Complete follow up | **Can’t Tell** |
| 7: Similarity in outcome measurement | **Yes** |
| 8: Reliability of outcome measurement | **Yes** |
| 9: Appropriate statistical analysis | **Yes** |
| TOTAL | **8** |

| Critical Appraisal Framework for Original Research (CAFFOR) checklist for Mixed Method study | | | | |
| --- | --- | --- | --- | --- |
| Individual study | **Kaphle et al., 2015** | **Ward et al., 2020** | **Ward et al., 2021** | |
| 1: Research problem, purpose, objectives, question, and rationale | **Yes** | **Yes** | | **Yes** |
| 2: Research approach and study design | **Yes** | **Yes** | | **Yes** |
| 3: Population, sampling, and sample size | **No** | **Yes** | | **No** |
| 4: Ethical conduct | **Yes** | **Yes** | | **Yes** |
| 5: Recruitment, participation, data collection | **Yes** | **Yes** | | **Yes** |
| 6: Interpreting key findings | **Yes** | **Yes** | | **Yes** |
| 7: Contribution and transferability | **Yes** | **Yes** | | **Yes** |
| TOTAL | **6** | **7** | | **6** |
