# Supplementary figures and images for: Use of mHealth in promoting maternal and child health in “BIMARU” states of India “A health system strengthening strategy”: Systematic literature review
Source: PLOS Digit Health. 2024 Feb 2;3(2):e0000403. doi: 10.1371/journal.pdig.0000403 (PMC10836675; doi:10.1371/journal.pdig.0000403)

S2 Appendix

***
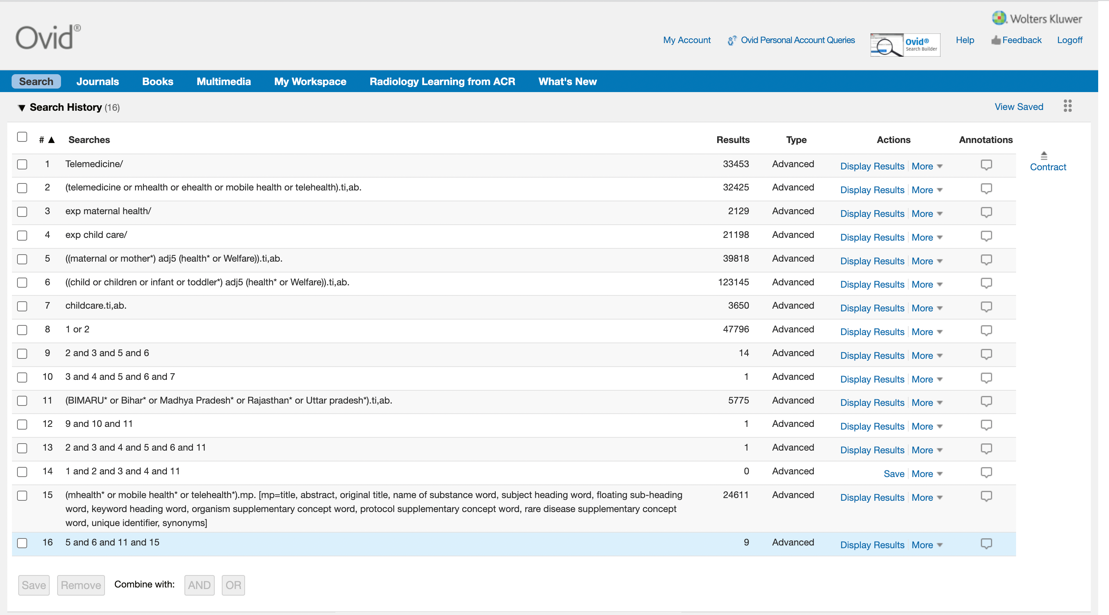
***


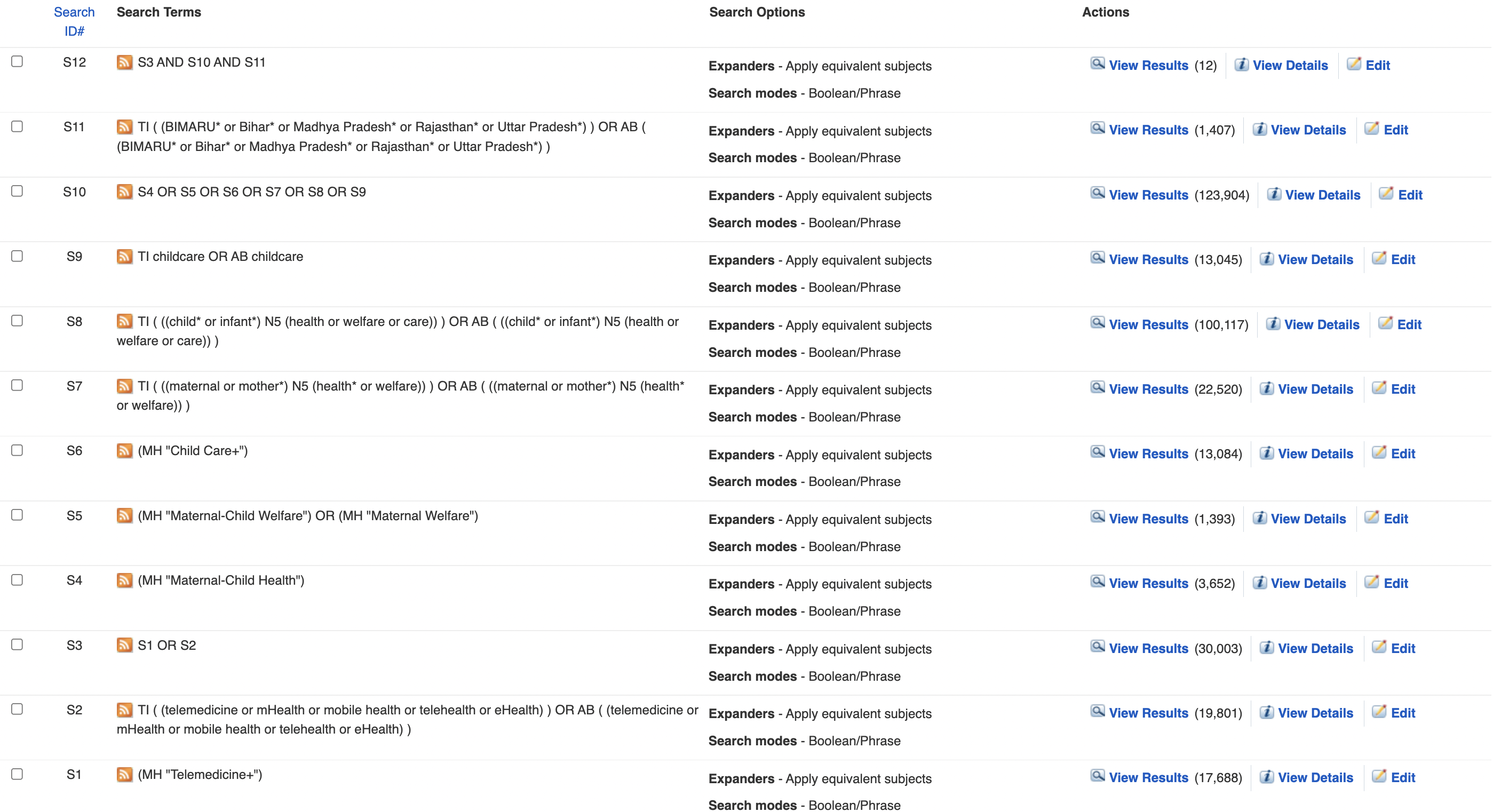


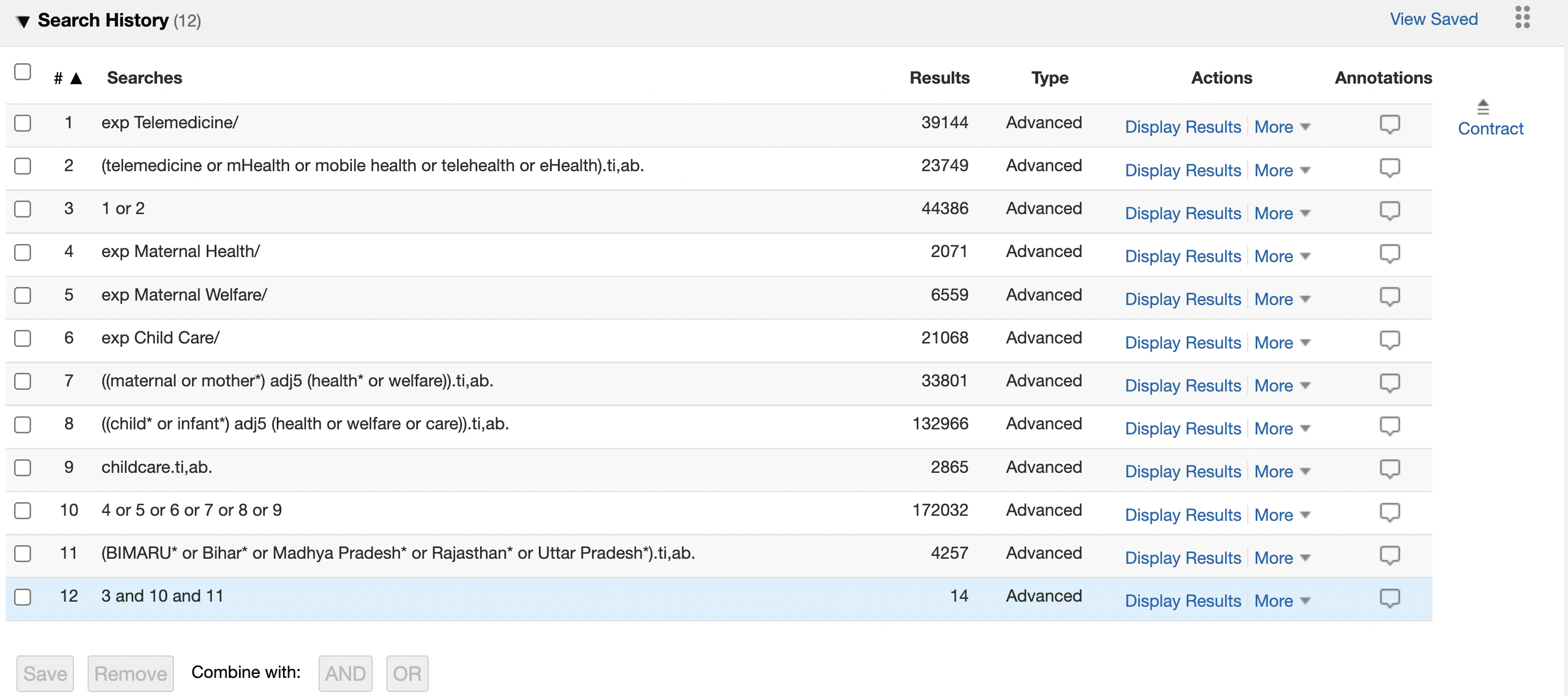


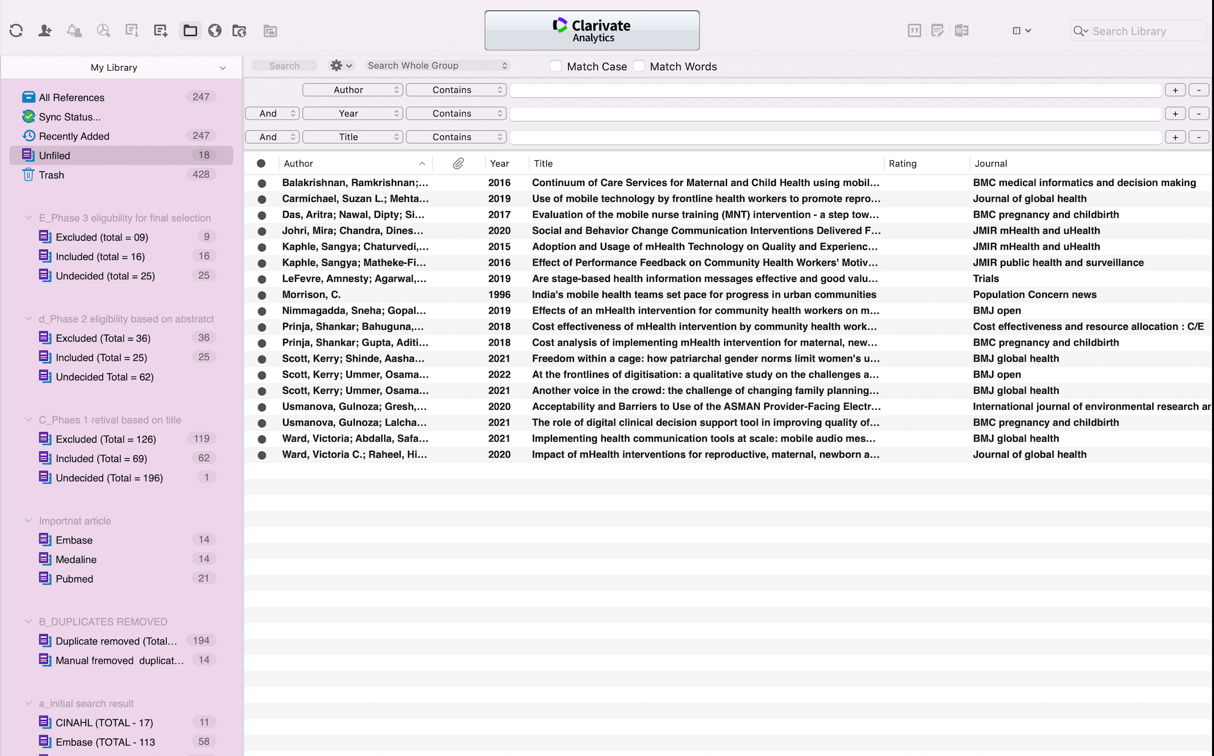

Supplement: S2 Appendix — (DOCX) [file pdig.0000403.s003.docx]
