# Supplementary material for: Use of mHealth in promoting maternal and child health in “BIMARU” states of India “A health system strengthening strategy”: Systematic literature review
Source: PLOS Digit Health. 2024 Feb 2;3(2):e0000403. doi: 10.1371/journal.pdig.0000403 (PMC10836675; doi:10.1371/journal.pdig.0000403)
Supplement: S1 Text — (DOCX) [file pdig.0000403.s004.docx]

**SEARCH STRING OF EACH DATABASE**

**1^st^ Database CINALH**

| **Search ID** | **Search Term** | **Action/result** |
| --- | --- | --- |
| S1 | (MH "Telemedicine+") | (17,688) |
| S2 | TI ( (telemedicine or Health or mobile health or telehealth or Health) ) OR AB ( (telemedicine or subjects mHealth or mobile health or telehealth or Health) ) | (19,801) |
| S3 | S1 OR S2 | (30,003) |
| S4 | (MH "Maternal-Child Health") | (3,652) |
| S5 | (MH "Maternal-Child Welfare") OR (MH "Maternal Welfare") | (1,393) |
| S6 | (MH "Child Care+") | (13,084) |
| S7 | TI (((maternal or mother*) N5 (health* or welfare))) OR AB (((maternal or mother*) N5 (health*or welfare))) | (22,520) |
| S8 | TI (((child* or infant*) N5 (health or welfare or care))) OR AB (((child* or infant*) N5 (health or welfare or care))) | (100,117) |
| S9 | TI childcare OR AB childcare | (13.045) |
| S10 | S4 OR S5 OR S6 OR S7 OR S8 OR S9 | (123,904) |
| S11 | ((BIMARU* or Bihar* or Madhya Pradesh* or Rajasthan* or Uttar Pradesh*)) OR AB ((BIMARU* or Bihar* or Madhya Pradesh* or Rajasthan* or Uttar Pradesh*)) | (1,407) |
| S12 | S3 AND S10 AND S11 | (17) |

2^nd^ Database **Embase**

| Sr No | Searches | Results |
| --- | --- | --- |
| 1 | exp Telemedicine/ | 39144 |
| 2 | (telemedicine or Health or mobile health or telehealth or Health).ti,ab. | 23749 |
| 3 | 1 or 2 | 44386 |
| 4 | exp Maternal Health/ | 2071 |
| 5 | exp Maternal Welfare/ | 6559 |
| 6 | exp Child Care/ | 21068 |
| 7 | ((maternal or mother*) adi5 (health* or welfare)) ti ab. | 33801 |
| 8 | (child* or infant*) ad5 (health or welfare or care)).ti.ab. | 132966 |
| 9 | childcare. ti.ab. | 2865 |
| 10 | 4or5 or 6 or 7 or 8 or 9 | 172032 |
| 11 | (BIMARU* or Bihar* or Madhya Pradesh* or Rajasthan* or Uttar Pradesh*).ti.ab. | 4257 |
| 12 | 3 and 10 and 11 | 113 |

3^rd^ **Database Medline**

| Sr No. | Searches | Results |
| --- | --- | --- |
|  | (health or "mobile health**).mp. [mp=title, abstract, original title, name of substance word, subject heading word, floating sub-heading word, keyword heading word, organism supplementary concept word, protocol supplementary concept word, rare disease supplementary concept word, unique identifier, synonyms] | 14811 |
|  | Telehealth.mp. or Telemedicine/ | 38047 |
|  | 1 or 2 | 48410 |
|  | (child* or infant* or (health* or care* or welfare*).mp. [mp=title, abstract, original title, name of substance word, subject heading word, floating subheading word, keyword heading word, organism supplementary concept word, protocol supplementary concept word, rare disease supplementary concept word, unique identifier, synonyms] | 7935038 |
|  | (child* or infant* or (health* or care* or welfare*\|).ti.ab. | 5655942 |
|  | (maternal or mother* or (welfare* or health* or care*). ti,ab. | 4645942 |
|  | (BIMARU* or Rajasthan* or Uttar Pradesh* or Madhya Pradesh* or Bihar*).mp. [mp=title, abstract, original title, name of substance word, subject heading word, floating sub-heading word, keyword heading word, organism supplementary concept word, protocol supplementary concept word, rare disease supplementary concept word, unique identifier, synonyms] | 5784 |
|  | 4 or 5 or 6 | 8080126 |
|  | 3 and 7 and 8 | 36 |
|  | India.mp. or India/ | 169752 |
|  | 3 and 8 and 10 | 605 |
|  | 1 and 7 and 8 and 10 | 106 |

**4^th^ Data Base PubMed**

| Sr No | Searches | Results |
| --- | --- | --- |
|  | **((((maternal or mother*) AND (health* or welfare)) AND (((child*) or (infant)) AND ((health*) OR (welfare)))) AND (((((Telehealth) OR (mobile health)) OR (telemedicine)) OR (exp telemedicine)) OR (mHealth))) AND ((((((Bihar) OR (Rajasthan)) OR (Uttarpradesh)) OR (Uttar Pradesh)) OR (Madhyapradesh)) OR (Madhya Pradesh))** | 42 |
|  | **(((((Bihar) OR (Rajasthan)) OR (Uttarpradesh)) OR (Uttar Pradesh)) OR (Madhyapradesh)) OR (Madhya Pradesh)** | 45,463 |
|  | **(((maternal or mother*) AND (health* or welfare)) AND (((child*) or (infant)) AND ((health*) OR (welfare)))) AND (((((Telehealth) OR (mobile health)) OR (telemedicine)) OR (exp telemedicine)) OR (mHealth))** | 2,237 |
|  | **((maternal or mother*) AND (health* or welfare)) AND (((child*) or (infant)) AND ((health*) OR (welfare)))** | 223,237 |
|  | **(Maternal or mother*) AND (health* or welfare)** | 300,940 |
|  | **((child*) or (infant)) AND ((health*) OR (welfare))** | 1,241,154 |
|  | **(health*) OR (welfare)** | 7,324,332 |
|  | **(child*) or (infant)** | 3,716,878 |
|  | **(maternal) OR (or mother)** | 605,148 |
|  | **((((Telehealth) OR (mobile health)) OR (telemedicine)) OR (exp telemedicine)) OR (mHealth)** | 107,907 |
